# Intraoperative choroidal detachment during small-gauge vitrectomy: analysis of causes, anatomic, and visual outcomes

**DOI:** 10.1038/s41433-021-01605-y

**Published:** 2021-06-21

**Authors:** Ting Zhang, Yantao Wei, Zhaotian Zhang, Wei Chi, Lujia Feng, Wu Xiang, Li Wang, Dong Fang, Yunhong Shi, Shaochong Zhang

**Affiliations:** 1 State Key Laboratory of Ophthalmology, Zhongshan Ophthalmic Center, Sun Yat-Sen University, Guangzhou, China; 2 Shenzhen Eye Hospital, Shenzhen Eye Institute, Jinan University, Shenzhen, China

**Keywords:** Uveal diseases, Diseases

## Abstract

**Introduction:**

To investigate the incidence and causes of intraoperative choroidal detachment (CD) during small-gauge vitrectomy, as well as the anatomic and visual outcomes.

**Methods:**

We retrospectively reviewed the medical records of 1026 consecutive patients who underwent small-gauge vitrectomy from June 2017 to December 2018 at Zhongshan Ophthalmic Centre, Guangzhou, China. Data on the presence, location, and extent of intraoperative CD and its relationship to the infusion cannula were collected. Patient demographic characteristics and postoperative anatomic and visual outcomes were also assessed.

**Results:**

A total of six cases were found to have intraoperative CD, including two with serous CD, three with limited haemorrhagic CD, and one with CD caused by inadvertent perfusion of gas during air/fluid exchange. Retraction of the infusion cannula and acute ocular hypotony were found to be the main causes of intraoperative CD in five out of the six cases. The best-corrected visual acuity of all cases significantly improved after the surgery.

**Conclusion:**

The incidence of intraoperative CD during small-gauge vitrectomy is low; the predominant causes are retraction of the infusion cannula and acute ocular hypotony. Immediate awareness and timely closure of the incision may contribute to a better surgical prognosis.

## Introduction

Pars plana vitrectomy (PPV) has been widely acknowledged as an effective and safe surgical intervention for a wide range of vitreoretinal diseases [1, 2]. Nowadays, small-gauge vitrectomy has been more widely used than the standard 20-gauge (20-G) PPV due to its well-reported advantages. In a small-gauge vitrectomy, sclerotomies are created with trocars to place the cannulas across the conjunctiva, sclera, and pars plana [3–9]. Although the safety of small-gauge vitrectomy has been widely recognised, intraoperative choroidal detachment (CD) occasionally occurs and may cause loss of visual acuity or even result in ocular enucleation as an expulsive choroidal haemorrhage [10, 11]. The intraoperative CD is an uncommon but devastating complication of PPV, which could severely worsen the patient’s surgical prognosis if there is not an appropriate intervention [12–14]. It is thought that intraoperative hypotony and inflammation are the main precipitating factors, causing alterations to transluminal vascular pressure. In addition, infusion cannula retraction is an important cause of the development of intraoperative CD during 23-G PPV [15–19]. At present, the appropriate timing of surgical drainage of CD is controversial, and in most cases, it is not associated with a better outcome.

To the best of our knowledge, intraoperative CD has not been well documented in terms of the cause, management and prognosis because of its minimal incidences. However, it is urgent to summarise the characteristics of the intraoperative CD to guide clinical treatment. Therefore, we investigated the incidence of intraoperative CD during small-gauge PPV; analysed the causes; and observed the long-term outcomes of the patients.

## Methods

This study was approved by the Institutional Review Board of Zhongshan Ophthalmic Centre (ZOC), affiliated with Sun Yat-sen University, Guangzhou, China, and performed in accordance with the World Medical Association’s Declaration of Helsinki. The medical records were retrospectively reviewed of 1026 consecutive inpatients who underwent standard three-port small-gauge PPV (including 23-G, 25-G, and 27-G) from 1 June 2017 to 31 December 2018, at ZOC of Sun Yat-sen University. Patients with a postoperative follow-up shorter than 3 months were excluded.

All the patients underwent small-gauge PPV (Constellation Vitrectomy System, Alcon Laboratories Fort Worth, TX) with retrobulbar anaesthesia by the same experienced ophthalmologist (SZ) under monitored anaesthesia care. A trocar cannula incision was created at 3.5 mm and 4 mm posterior to the limbus in pseudophakic/aphakic and phakic eyes, respectively. The trocar cannula incision was created with a straight (perpendicular to the sclera) or angled (15- to 45-degree angle to the sclera) approach, according to the surgeon’s preference and judgment. An infusion cannula was placed in the inferotemporal quadrant. Appropriate placement of the infusion cannula into the vitreous cavity was visually confirmed before the infusion was administered, and the infusion line was attached with a surgical drape. Standard PPV was performed in all the cases. After core vitrectomy, the assistants performed peripheral vitrectomy with a scleral indentation in the patients where it was deemed necessary. Laser endophotocoagulation was used in cases with a retinal tear, retinal detachment, or diabetic retinopathy. Tamponade agents (fluid, air, long-acting gas, or silicone oil) were chosen and applied according to each patient’s individual vitreoretinal condition. After cannula removal, all sclerotomy sites were inspected to confirm there was no obvious leakage, and if necessary, sutures were placed to prevent leakage. Statistical analysis was performed using STATA software (version 14.0; STATA Corp., College Station, TX, USA). A *P* value less than 0.05 was considered statistically significant.

## Results

### Clinical results

**Fig. 1:**
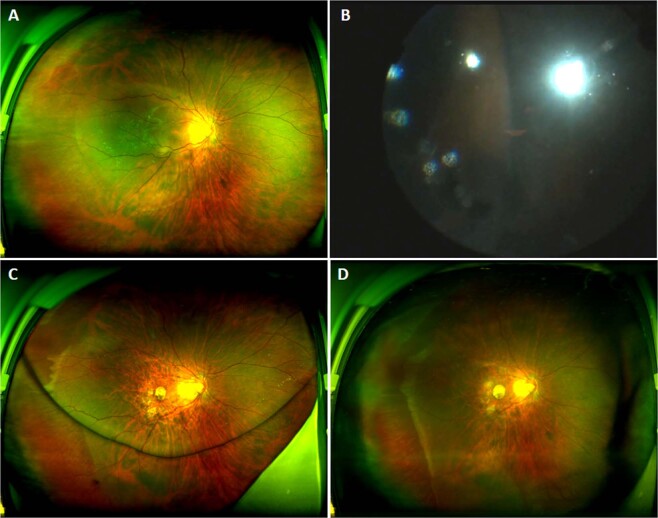
Ultra-widefield images of the left eye of patient 3. **A** Pre-operation of the macular hole with retinal detachment. **B** Haemorrhagic choroidal detachment was observed from the temporal quadrant at the time of silicone oil filling during 25 G PPV. **C** One-month post-operation of PPV and silicone oil tamponade, the retina was flat. **D** One-month post-operation of silicone oil removed.

**Fig. 2:**
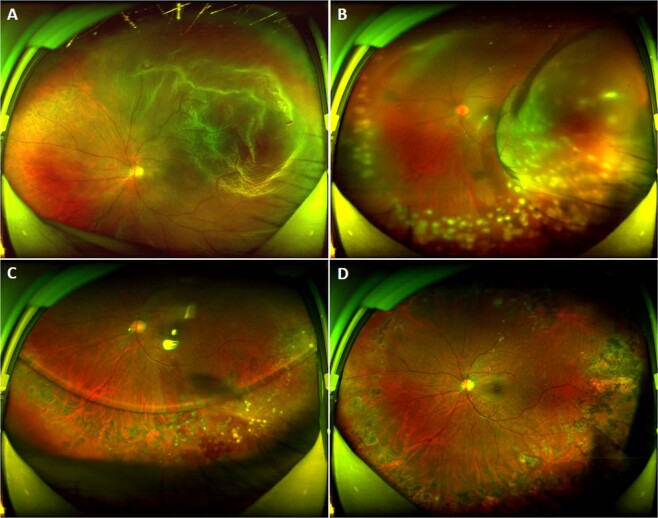
Ultra-widefield images of the left eye of patient 5. **A** Pre-operation of rhegmatogenous retinal detachment. **B** One day post-operation of PPV and silicone oil tamponade: haemorrhagic choroidal detachment was observed at 1–5 o’clock position. **C** One-month post-operation of PPV and silicone oil tamponade, the retina was flat. **D** One-month post-operation of silicone oil removed.

A total of 1026 consecutive patients were included in the final analysis, of whom six developed intraoperative CD. These six CDs contained serous fluid in two, blood in three, and C3F8 in one. The mean age of the six patients was 51.6 ± 13.7 years (range, 33–75 years), and 50% were female. The surgical indication for PPV was a macular hole with retinal detachment in one case (Patient 3) and rhegmatogenous retinal detachment (RRD) in the other five cases (Patients 1, 4–6). In all cases with CD, the detachment originated at the infusion cannula site. CD was limited to three clocks in four cases, and the other two had the involvement of six clocks. Secondary retinal apposition or expulsion of intraocular contents was not observed in all cases. One case of serous CD (SCD) and one of haemorrhagic CD (HCD) extended to the submacular region. Two typical fundus photographs and operation videos of the cases (Patients 3 and 5) are provided in Figs. 1 and 2 and Video 1 respectively.

Infusion cannula retraction and subsequent hypotony were noted in five of the six cases of CD (Patients 1, 2, 4, 5, and 6). In the one remaining case of HCD (Patient 3), the infusion cannula retraction was not observed. However, Patient 3 had multiple risk factors for CD, including high myopia (<−10 dioptres), posterior staphylomas, macular hole, and retinal detachment. In another case of HCD (Patient 5), removal and replacement of the infusion tube into one of the superior cannulas with the subsequent creation of a new inferonasal infusion resulted in immediate intraoperative stabilisation. All the patients had at least 3 months of follow-up, with a mean postoperative follow-up duration of 10.60 ± 4.88 months. The intraocular pressure was within the normal range (10–21 mmHg) for all patients, and surgical intervention was unnecessary throughout the follow-up. Anatomic success (flat retina) was achieved by a median follow-up of 11 months (range, 3–17 months). At the final follow-up, all the patients achieved significant improvement of BCVA (*P* < 0.05). A final visual acuity of 20/25 or greater was observed in two patients, with a BCVA of 20/16 in one patient, and only one patient had a final visual acuity of counting fingers. All cases of CD spontaneously resolved in the postoperative period without intervention, where a preoperative status such as with the visual acuity of Patient 4, having an intact macula before the operation, had the best prognosis (20/16) (Table 1).

**Table 1:** Demographic and clinical characteristics of the six cases with choroidal detachment.

Case no.	Age, years	Sex	Surgical indication	Surgical procedure	Time point of CD occurrence	Choroidal detachment	Development at cannula site	Hypotony	Cannula status	Results of fundus examination at the end of the operation	Pre-/post-operative and final visual acuity	Follow-up duration (Months)	Cause analysis
1	61	F	RRD	25GPPV, AFE, EL, SOT	During AFE	Serous	Yes	Yes	Retracted	Good red reflex; Mild SCD	20/2000; HM/50 cm; 20/1000	3	RRD, thin and poor elasticity of sclera, deep eyehole, tight lids
2	75	F	RRD	23GPPV, AFE, EL, SOT	During AFE	Haemorrhagic	Yes	Yes	Retracted	Poor red reflex; Large HCD; VH	HM/30 cm; HM/30 cm; HM/50 cm	6	RRD, advanced age
3	53	F	MH, RRD, high myopia	25GPPV, AFE, EL, SOT	During SOT	Haemorrhagic	Yes	No	Neither	Good red reflex; Mild HCD	CF/50 cm; 20/1000; 20/1000	13	RRD, high myopia
4	36	M	RRD	27GPPV, AFE, EL	During AFE	C3F8	Yes	Yes	Retracted	Good red reflex; Mild CD	CF/30 cm; CF/30 cm; 20/16	17	RRD, myopia
5	33	M	RRD, high myopia	25GPPV, AFE, EL, SOT	During AFE	Haemorrhagic	Yes	Yes	Retracted	Good red reflex; Large	CF/30 cm; CF/30 cm; 20/25	14	RRD, high myopia
6	60	F	RRD	25GPPV, AFE, EL	During AFE	Serous	Yes	Yes	Retracted	HCD; Good red reflex; Mild SCD	HM/30 cm; HM/50 cm; 20/200	3	RRD

*F* female, *M* male, *RRD* rhegmatogenous retinal detachment, *MH* macular hole, *23G* 23-gauge, *25G* 25-gauge, *27G* 27-gauge, *PPV* pars plana vitrectomy, *AFE* air-fluid exchange, *EL* endolaser, *SOT* silicone oil tamponade, *SCD* serous choroidal detachment, *HCD* haemorrhagic choroidal detachment, *VH* vitreous haemorrhage, *VA* visual acuity, *HM* hand motion, *CF* counting fingers.

## Discussion

Intraoperative CD, especially suprachoroidal haemorrhage, is an uncommon but dangerous complication during various intraocular surgeries, and CD usually leads to poor visual acuity or even phthisis [20–23]. According to the different components, intraoperative CD can be serous, haemorrhagic, gas, or even oily [9]. Currently, small-gauge vitrectomies are widely used for retinal diseases due to their multiple advantages, which include more rapid visual recovery, reduced conjunctival scarring, and decreased postoperative inflammation [8, 24]. This study described the incidence of CD during 23-G, 25-G, and 27-G PPV [25, 26], suggesting that cannula retraction and acute ocular hypotony may be the main causes of intraoperative CD during small-gauge PPV.

The incidence of CD has been reported during 20-G and 23-G PPV [27–30], where the incidence of SCDs was 0.4–0.5% and 0–1.8% in 20-G PPV and 23-G PPV, respectively [31–34]. In our study, two cases of SCD were identified out of 1026 consecutive small-gauge vitrectomies, resulting in an incidence of 0.195%, which is lower than that reported by Tarantola et al. [29] during 23-G PPV (1.77%). The rate of HCD during 20-G PPV was reported in previous studies as varying from 0.17% to as high as 1.9% [35, 36]. Zhang et al. [37] demonstrated that 23G PPV has a lower risk of choroidal haemorrhage than 20-G vitrectomy particularly for eyes with RRD. Tarantola et al. [29] reported four cases of HCD during 338 consecutive 23-G PPVs (1.18%). Sporadic cases of HCD during small-gauge vitrectomies have been reported [25, 26]; however, the incidence of HCD during 25-G PPV and 27-G PPV have not been reported. In our study, we reported three cases of HCD out of 1026 consecutive small-gauge vitrectomies, resulting in an incidence of 0.029%, which is significantly lower than that reported in 20-G PPV and 23-G PPV [29]. The lower incidence of SCD and HCD in our study could be attributed to the following reasons. First, extensive preoperative CD associated with RRD is an unfavourable condition for surgical intervention, and we have reported a systematic approach, which was used in all the cases in our study, to assist in the safe placement of trocar cannulas, thus preventing inadvertent fluid/air perfusion during PPV in RRD eyes with extensive CD, suprachoroidal fluid, and hypotony [37]. Second, other important aspects include insertion of the trocar at a 90-degree angle rather than an oblique angle, which creates an orthographic wound. A method previously reported and used in our cases involves visual verification after insertion of at least 2 mm of the infusion cannula into the vitreous cavity and securing the infusion line with Steri-Strips to the surgical drape so that the cannula is oriented at 90 degrees to the sclera.

Cause analyses for the development of CD during small-gauge PPV are not yet clear. Reported causes for CD during 20-G PPV include ocular hypotony, choroidal vascular instability generated by arterial hypertension, and increased central venous pressure generated by the Valsalva manoeuvre [11, 38]. In a series of four HCD cases during 23-G PPV, Tarantola et al. [29] reported that the risk factors were high levels of myopia, previous retinal detachment surgery, presence of an RRD, encircling with a broad sclera buckle, cryotherapy, external transchoroidal drainage of subretinal fluid, and intraoperative systemic hypertension. However, in this study, it has been suggested that infusion cannula retraction and acute ocular hypotony may be the most important mechanisms and risk factors. The evidence for this suggestion includes the following: all our patients experienced some hypotony; in two cases of SCD and two of the three HCD cases, the infusion cannula was visibly repeatedly retracted, and the location of the CD in all of these cases was the same quadrant as the infusion cannula; and the time point of SCD was at the beginning of the air/fluid exchange (AFE), suggesting that the direct infusion of fluid into the suprachoroidal space before the air infusion resulted in this complication.

In addition, the hypothesis that cannula retraction is responsible for SCD is further supported by cases of direct infusion of air through a cannula that was retracted into the suprachoroidal space, resulting in a localised CD. Furthermore, when scleral indentation is performed by an inexperienced assistant to remove the peripheral vitreous near the infusion cannula, the unsutured small-gauge cannula in a shallow bevelled sclerotomy may be retracted beneath the pars plana. Moreover, thin and poor elasticity of the sclera, a deep eyehole, and tight lids will all facilitate cannula retraction. Additionally, intraoperative intraocular pressure fluctuations may be another cause for the development of intraoperative CD during small-gauge vitrectomy. It is worth noting that four of the six-CD cases occurred at the time point of AFE, and one case occurred at the time of silicone oil filling. The higher risk of CD at the beginning of air/liquid exchange or oil/air exchange could be partly explained by two factors: (1) the intraocular pressure fluctuates with a large suction force, and the air/oil perfusion pressure is not able to maintain normal intraocular pressure; and (2) when the intraocular pressure drops, a trocar without valves is more likely to retract into the suprachoroidal space.

Intraoperative suprachoroidal haemorrhage has consistently resulted in poor vision in previously published cases. Lakhanpal et al. [39] reported that six of seven patients with HCD during PPV had a final visual acuity of no light perception. Chandra et al. [30] showed that 29% of cases of HCD achieved counting fingers or hand motion, and the remainder (17.1%) only had a perception of light or worse. It is of interest that in this study, all the patients achieved anatomic success and significant improvement of BCVA (even 20/25 or greater in two patients) at the final follow-up. Obviously, the visual acuity prognosis of our cases was better than in previous studies. The reasons for the satisfactory anatomic and visual outcomes include the incidence of HCD accounting for only three out of six cases; timely awareness of CD during the operation; and immediate suturing of all surgical incisions, which certainly may help with better vision recovery. The details of how we managed this accidental complication during the surgery are as follows:

1. When there was awareness of CD, we quickly attached the infusion tube to one of the superior cannulas to re-establish intraocular pressure, and we observed that the SCD resolved immediately in two cases.
2. Had a short communication with our patients to calm down their nervousness and asked for their full cooperation during the whole surgical procedure.
3. Thoroughly checked the infusion line and the infusion trocar, figured out the real underlying causes of the accidental condition.
4. Considered whether or not to continue the following surgical procedure, based on the severity of the CD.
5. If not to continue, we would explain the real conditions to the patient, seal all three incisions and have a strict observation of the CD for several days until it was decided that a second surgery was permittable to eliminate the CD and to achieve retinal re-attachment. It is very important to close all surgical incisions immediately, which was done in our cases, so the scope of CD in our cases was limited.
6. If to continue, we would create infusion at another new safe place, such as inferonasal infusion, or use a longer infusion trocar-cannula, in order to assure adequate fluid infusion into the vitreous cavity.
7. Once safe infusion was achieved by the above methods, we continued the following surgical manipulations as routine, such as perfluorocarbon liquid infusion, laser photocoagulation, fluid/air exchange, silicone oil infusion, and finally to make retinal re-attachment.
8. Finally, we sealed the incisions and have strict monitoring of the patients’ intraocular pressure and the progression of the CD after the surgery.

Although we have taken many measures to prevent hypotony during small-gauge vitrectomy, intraoperative CD still occasionally happens. Steps that can be taken to help avoid these harmful events may include placing the infusion tube on the palpebral fissure as far as possible when the eyelid is tight and the eye socket is deep; alerting assistants to avoid movements that may retract the infusion line; periodically confirming the external appearance of the infusion cannula during surgery to recognise any retraction; visually rechecking the cannula position before injection of gas or silicone oil into the eye; and bearing in mind that intraoperative retraction and acute ocular hypotony may occur, particularly during scleral depression.

## Conclusions

In summary, cannula retraction and acute ocular hypotony are the predominant causes for the development of CD during small-gauge PPV. Predictors of a good visual outcome include shorter duration of macular detachment, preoperative prevention, immediate recognition during the operation, and instant and limited intervention.

### Summary

#### What was known before

- Although the safety of small-gauge vitrectomy has been widely recognised, intraoperative choroidal detachment (CD) occasionally occurs and may cause loss of visual acuity or even result in ocular enucleation as an expulsive choroidal haemorrhage.

#### What this study adds

- The incidence of intraoperative CD during small-gauge vitrectomy is low; the predominant causes are retraction of the infusion cannula and acute ocular hypotony. Immediate awareness and timely closure of the incision may contribute to a better surgical prognosis.

## Supplementary information

Video 1

## References

[1] Mendrinos E, Dang-Burgener NP, Stangos AN, Sommerhalder J, Pournaras CJ (2008). Primary vitrectomy without scleral buckling for pseudophakic rhegmatogenous retinal detachment. Am J Ophthalmol.

[2] Ahmadieh H, Moradian S, Faghihi H, Parvaresh MM, Ghanbari H, Mehryar M (2005). Anatomic and visual outcomes of scleral buckling versus primary vitrectomy in pseudophakic and aphakic retinal detachment: six-month follow-up results of a single operation−report no. 1. Ophthalmology.

[3] Lakhanpal V, Schocket SS, Elman MJ, Dogra MR (1990). Intraoperative massive suprachoroidal hemorrhage during pars plana vitrectomy. Ophthalmology.

[4] Inoue Y, Kadonosono K, Yamakawa T, Uchio E, Watanabe Y, Yanagi Y (2009). Surgically-induced inflammation with 20-, 23-, and 25-gauge vitrectomy systems: an experimental study. Retina.

[5] Woo SJ, Park KH, Hwang JM, Kim JH, Yu YS, Chung H (2009). Risk factors associated with sclerotomy leakage and postoperative hypotony after 23-gauge transconjunctival sutureless vitrectomy. Retina.

[6] Kim YK, Hyon JY, Woo SJ, Park KH, Yu YS, Chung H (2010). Surgically induced astigmatism after 23-gauge transconjunctival sutureless vitrectomy. Eye.

[7] Nagiel A, McCannel CA, Moreno C, McCannel TA (2017). Vitrectomy-assisted biopsy for molecular prognostication of choroidal melanoma 2 mm or less in thickness with a 27-gauge cutter. Retina.

[8] Khan MA, Kuley A, Riemann CD, Berrocal MH, Lakhanpal RR, Hsu J (2018). Long-term visual outcomes and safety profile of 27-gauge pars plana vitrectomy for posterior segment disease. Ophthalmology.

[9] Lin CJ, Peng KL (2018). Intraoperative severe suprachoroidal air as a complication of 23-gauge vitrectomy combined with air-fluid exchange. Int Med Case Rep J.

[10] Tabandeh H, Sullivan PM, Smahliuk P, Flynn HJ, Schiffman J (1999). Suprachoroidal hemorrhage during pars plana vitrectomy. Risk factors outcomes. Ophthalmology.

[11] Tabandeh H, Flynn HJ (2001). Suprachoroidal hemorrhage during pars plana vitrectomy. Curr Opin Ophthalmol.

[12] Welch JC, Spaeth GL, Benson WE (1988). Massive suprachoroidal hemorrhage. Follow-up and outcome of 30 cases. Ophthalmology.

[13] Payne JW, Kameen AJ, Jensen AD, Christy NE (1985). Expulsive hemorrhage: its incidence in cataract surgery and a report of four bilateral cases. Trans Am Ophthalmol Soc.

[14] Taylor DM (1975). Letter: expulsive hemorrhage. Am J Ophthalmol.

[15] Lopez-Guajardo L, Pareja-Esteban J, Teus-Guezala MA (2006). Oblique sclerotomy technique for prevention of incompetent wound closure in transconjunctival 25-gauge vitrectomy. Am J Ophthalmol.

[16] Taban M, Ventura AA, Sharma S, Kaiser PK (2008). Dynamic evaluation of sutureless vitrectomy wounds: an optical coherence tomography and histopathology study. Ophthalmology.

[17] Singh RP, Bando H, Brasil OF, Williams DR, Kaiser PK (2008). Evaluation of wound closure using different incision techniques with 23-gauge and 25-gauge microincision vitrectomy systems. Retina.

[18] Taban M, Sharma S, Ventura AA, Kaiser PK (2009). Evaluation of wound closure in oblique 23-gauge sutureless sclerotomies with visante optical coherence tomography. Am J Ophthalmol.

[19] Gupta OP, Maguire JI, Eagle RJ, Garg SJ, Gonye GE (2009). The competency of pars plana vitrectomy incisions: a comparative histologic and spectrophotometric analysis. Am J Ophthalmol.

[20] Chu TG, Cano MR, Green RL, Liggett PE, Lean JS (1991). Massive suprachoroidal hemorrhage with central retinal apposition. A clinical and echographic study. Arch Ophthalmol.

[21] Speaker MG, Guerriero PN, Met JA, Coad CT, Berger A, Marmor M (1991). A case-control study of risk factors for intraoperative suprachoroidal expulsive hemorrhage. Ophthalmology.

[22] Palamar M, Thangappan A, Shields CL, Ehya H, Shields JA (2009). Necrotic choroidal melanoma with scleritis and choroidal effusion. Cornea.

[23] Mei H, Xing Y, Yang A, Wang J, Xu Y, Heiligenhaus A (2009). Suprachoroidal hemorrhage during pars plana vitrectomy in traumatized eyes. Retina.

[24] Jiang X, Zhang S, Zhang Z, Zhou X, Wei Y (2018). Comparative study of 27-gauge versus 25-gauge vitrectomy with air tamponade in the treatment of myopic foveoschisis. Ophthal Surg Lasers Imaging Retin.

[25] Kapamajian M, Gonzales CR, Gupta A, Schwartz SD (2007). Suprachoroidal hemorrhage as an intraoperative complication of 25-gauge pars plana vitrectomy. Semin Ophthalmol.

[26] Chen CJ, Satofuka S, Inoue M, Ishida S, Shinoda K, Tsubota K (2008). Suprachoroidal hemorrhage caused by breakage of a 25-gauge cannula. Ophthalmic Surg Lasers Imaging.

[27] Ooto S, Kimura D, Itoi K, Mukuno H, Kusuhara S, Miyamoto N (2008). Suprachoroidal fluid as a complication of 23-gauge vitreous surgery. Br J Ophthalmol.

[28] Chieh JJ, Rogers AH, Wiegand TW, Baumal CR, Reichel E, Duker JS (2009). Short-term safety of 23-gauge single-step transconjunctival vitrectomy surgery. Retina.

[29] Tarantola RM, Folk JC, Shah SS, Boldt HC, Abràmoff MD, Russell SR (2011). Intraoperative choroidal detachment during 23-gauge vitrectomy. Retina.

[30] Chandra A, Xing W, Kadhim MR, Williamson TH (2014). Suprachoroidal hemorrhage in pars plana vitrectomy: risk factors and outcomes over 10 years. Ophthalmology.

[31] Gupta OP, Ho AC, Kaiser PK, Regillo CD, Chen S, Dyer DS (2008). Short-term outcomes of 23-gauge pars plana vitrectomy. Am J Ophthalmol.

[32] Lott MN, Manning MH, Singh J, Zhang H, Singh H, Marcus DM (2008). 23-gauge vitrectomy in 100 eyes: short-term visual outcomes and complications. Retina.

[33] Schweitzer C, Delyfer MN, Colin J, Korobelnik JF (2009). 23-Gauge transconjunctival sutureless pars plana vitrectomy: results of a prospective study. Eye.

[34] Stein JD, Zacks DN, Grossman D, Grabe H, Johnson MW, Sloan FA (2009). Adverse events after pars plana vitrectomy among medicare beneficiaries. Arch Ophthalmol.

[35] Piper JG, Han DP, Abrams GW, Mieler WF (1993). Perioperative choroidal hemorrhage at pars plana vitrectomy. a case-control study. Ophthalmology.

[36] Sharma T, Virdi DS, Parikh S, Gopal L, Badrinath SS, Mukesh BN (1997). A case-control study of suprachoroidal hemorrhage during pars plana vitrectomy. Ophthalm Surg Lasers.

[37] Zhang Z, Fang D, Peng M, Wei Y, Wang L, Fan S (2019). Systemic approach to prevent inadvertent perfusion in eyes with extensive choroidal detachment, suprachoroidal fluid, and hypotony during pars plana vitrectomy. Adv Ther.

[38] Pollack AL, McDonald HR, Ai E, Johnson RN, Dugel PU, Folk J (2001). Massive suprachoroidal hemorrhage during pars plana vitrectomy associated with Valsalva maneuver. Am J Ophthalmol.

[39] Lakhanpal V, Schocket SS, Elman MJ, Nirankari VS (1989). A new modified vitreoretinal surgical approach in the management of massive suprachoroidal hemorrhage. Ophthalmology.

